# Multileaf Faraday cup for beam energy verification in radiation therapy with ultra‐high dose‐rate electron beams and ion beams

**DOI:** 10.1002/mp.70362

**Published:** 2026-03-08

**Authors:** Christoph Makowski, Michael Deutsch, Claus‐Stefan Schmitzer, Andreas Schüller

**Affiliations:** ^1^ Physikalisch‐Technische Bundesanstalt (PTB) Braunschweig Germany; ^2^ EBG MedAustron Wiener Neustadt Austria

**Keywords:** beam energy determination, charged particle beam, FLASH radiotherapy, multileaf Faraday cup

## Abstract

**Background:**

In radiotherapy and particle therapy, the stability of the beam energy must be checked routinely during quality assurance. This is of particular importance when varying the dose rate via the beam intensity of the accelerator in order to study the FLASH effect when comparing irradiations with conventional and ultra‐high dose rates.

**Purpose:**

The standard method of energy verification based on the measurement of percent depth–dose curves in a water phantom is time‐consuming and in the case of ultra‐high dose‐rate beams, may also present a non‐negligible radiation protection problem.

**Methods:**

A compact, portable multileaf Faraday cup (MLFC) with 128 channels was developed for energy determination based on the measurement of depth–charge curves. Its design is optimized for clinical electron beams in a range between 3 and 25 MeV. The read‐out unit of the MLFC displays the beam energy in real time. The device was tested in strongly pulsed electron beams such as those present in irradiations with ultra‐high doses per pulse as well as in conventional clinical electron beams. The same detector was used for proof of concept in a single measurement campaign in pulsed beams of therapeutical protons (conventional dose rates) from a synchrotron source using a suitable range shifter. The MLFC was calibrated with monoenergetic electron beams as well as against depth–dose curves. Simulations were carried out for comparison.

**Results:**

The MLFC works well under ultra‐high pulse dose rate conditions as well as in conventional electron beams generated by a medical accelerator. Changes in the beam energy of below 20 keV (0.1% of 20 MeV) can be clearly identified by means of the MLFC. For proton beams, well‐defined peaks in the depth–charge curves are observed for each single synchrotron spill with a total charge of only about 1 nC. The energy values resulting from MC simulations of the measured MLFC data agree with the actual proton energies within 2%.

**Conclusions:**

The MLFC can be used for quick validation of the energy stability when carrying out experiments comparing electron beams of conventional and ultra‐high dose rates. The same device can be used in the pulsed ion beams from a synchrotron.

## 1 INTRODUCTION

For quality assurance (QA) in radiotherapy and particle therapy, the stability of the beam energy must be checked at regular intervals in routine clinical practice. In particle therapy with protons or carbon ions, the energy is crucial for the exact depth of the Bragg peak and thus for safe and effective treatment.^1^ For radiotherapy with high‐energy electrons, the energy determines the electron penetration depth and thus the dose at the depth of the target volume.^2^

In preclinical studies of so‐called FLASH irradiations,^3^ cell‐based assays or organs of animals are irradiated with electron^4^ or proton beams^5^ at both ultra‐high and conventional dose rates. Different research groups have observed reduced radiation‐induced toxicities in healthy tissue at ultra‐high dose rates compared to a conventional dose delivery while maintaining an equivalent tumor response. This sparing effect is called the FLASH effect. An overview of the status of this research field can be found in several review articles.^4^, ^5^, ^6^, ^7^, ^8^, ^9^ For a comparison of the two irradiation modalities, it is desirable for the conventional reference beam to be the same as at the ultra‐high dose‐rate irradiation (i.e., for the dose rate to be varied via the beam intensity without changing other parameters relevant to the dose delivery). If possible, the energy should be the same and constant for the duration of the experimental campaign. However, the prototype linear accelerators (LINACs) used for FLASH studies^10^, ^11^ and commercially available LINACs for ultra‐high dose‐rate electron beam generation as ElectronFlash (SIT, Aprilia, Italy), Mobetron (IntraOp, Sunnyvale, USA), and FLASHKNiFE (PMB ALCEN, Peynier, France) do not have an electromagnet for separation of a monoenergetic electron beam. Therefore, the beam energy of these machines changes depending on pulse duration^12^ and pulse current^10^, ^11^, ^13^, ^14^ due to the effect of beam loading.^13^ The energy is also very sensitive to changes in the RF power. It is recommended to check the energy stability during daily and weekly QA.^12^ If the energy changes unnoticed, the dose at the measurement depth changes. Differences in the dose response between ultra‐high and conventional dose rates at allegedly same dose could then be misinterpreted as a FLASH effect.

At ion beams the energy should also be checked daily and monthly with an instrument independently of the internal beam diagnostics of the accelerator in particular for FLASH^15^ when approaching the limits of the beam intensity to achieve ultra‐high dose rates.^16^

In clinical practice, energy is not measured directly. Instead, energy‐dependent Percent Depth‐Dose (PDD) curves (i.e., characteristic dose distributions in the beam direction in a water phantom) are recorded during QA.^17^ For electron beams, the half‐value depth $R_{50}$ (i.e., the depth at which the absorbed dose drops to 50% of its maximum value), is checked for constancy. For ion beams, the position of the pristine Bragg peak is verified, e.g., as the depth at 80% dose in the distal fall‐off region $R_{80}$. However, this regular QA process is time‐consuming and requires expensive equipment. Reference PDD curves are measured with a suitable detector (ionization chamber, diode, diamond) immersed in a sufficiently large water tank with a motorized positioning system to scan the dose distribution. However, setting up the water tank and scanning PDD curves by moving the detector along the beam axis is excessively time‐consuming. In the case of ultra‐high dose‐rate beams, the latter may also present a non‐negligible radiation protection problem.

For QA of therapeutic proton beams, alternative faster measurement methods have been established.^18^ For pencil beam scanning, significant time can be saved, since a PDD curve has to be recorded for many different energies according to the different depths of the scanned layers. To measure the range of ion beams efficiently, multilayer ionization chambers (MLIC) have been developed.^18^, ^19^, ^20^, ^21^ MLICs consist of many (32 up to 180) stacked independent plane‐parallel ionization chambers in series in order to measure the dose simultaneously at different depths. The electrodes and dedicated absorbing material in between neighboring ionization chambers mimic a water‐equivalent thickness. These detectors are practical for quickly checking the beam range and thus the energy stability. Commercially available devices are Giraffe and Zebra^22^, ^23^, ^24^ (IBA Dosimetry, Schwarzenbruck, Germany) and QUBE^25^ (De.Tec.Tor., Torino, Italy). It is important to note that current commercial MLIC detectors are vulnerable to saturation effect at ultra‐high dose rate. Hence, for QA of ion beams for FLASH, it is recommended to adapt the dose rate.^15^

For electron beams, it is uncommon to use MLICs for QA. In the 90s, however, there was a commercially available MLIC for consistency checks of electron energy from radiotherapy accelerators, the so‐called Geske monitor (type 3405, PTW Freiburg).^26^, ^27^ This device containing nine parallel plate ionization chambers that alternate with 5‐mm‐thick aluminum absorbers.^26^ Measurable beam energy changes ranging from 1.7% (at 20 MeV) to 15% (at 6 MeV).^28^ However, for electron beams with ultra‐high dose rate, MLIC cannot be used at all, because ionization chambers show significant nonlinearities at ultra‐high dose per pulse due to ion recombination effects.^29^, ^30^, ^31^ This issue is particularly prevalent in plane‐parallel ionization chambers with large electrode distances (>0.5 mm) and/or at low operational voltages (<500 V),^32^ which is the case for the available MLICs.

A further alternative method is to use a scintillator to record a complete percent depth–light (PDL) curve; here, a monolithic scintillator block can be observed by a camera^33^, ^34^ or a stack of optically decoupled plastic scintillator sheets can be read out by a large‐scale CMOS sensor.^35^ However, scintillators may exhibit significant nonlinear dependence between the light output and the dose deposition (light quenching effects); thus, the measured PDL must be corrected accordingly.^34^ A significant problem entailed by the use of ultra‐high dose‐rate irradiation is the fact that plastic scintillators suffer from radiation damage and may rapidly change their response.

The third alternative method is to exploit the fact that ions and electrons are charged particles and to measure a percent depth‐charge curve using a multileaf Faraday cup (MLFC).^36^, ^37^ An MLFC is a stack of many (30 to 512) metal plates separated from each other by thin insulating layers. Each conducting plate is connected to ground via a charge meter using a multichannel charge integrator,^36^, ^38^, ^39^, ^40^ or a multichannel amperemeter.^41^ Incoming ions or electrons stop within a certain plate and the corresponding channel collects the deposited charge. In this way, the differential fluence as a function of depth can be measured.

All ions of a monoenergetic ion beam have a similar range in matter according to their energy and thus deposit their charge in a strictly limited way in only a few plates. The resulting peak in the percent depth–charge curve can be calibrated against a Bragg–Peak range measurement.^42^ MLFCs are used in proton therapy research centers^38^, ^39^, ^40^, ^41^, ^42^, ^43^, ^44^, ^45^, ^46^ and at other applications of MeV ion beams.^47^, ^48^ In recent years, MLFCs for measuring the energy of ion beams have become commercially available: for example, the MLFC‐128^40^, ^42^ (Pyramid Technical Consultants, Waltham, USA) and the QEye (De.Tec.Tor., Torino, Italy).

In contrast to ion beams in matter, electrons experience large deflections in each collision; thus, because their tracks are not straight and differ from one other, they stop at different depths. Hence, monoenergetic electron beams exhibit a broad range distribution (i.e., the incoming charge is distributed over all MLFC plates up to the maximum range according to the primary energy). The shape of the depth–charge curve and the position of the maximum depends on the energy.^49^ If the electron energy from the accelerator is fix or if it is variable only within a relatively narrow range, then an MLFC with only two plates is feasible. The thickness of the front plate is chosen depending on the beam energy, allowing it to collect about half of the charge. The ratio of the charges collected from the front and rear plates is quite sensitive to the beam energy.^50^, ^51^, ^52^ Berne et al. developed a noninterceptive two‐plate MLFC for electron energy monitoring for a 6‐MeV LINAC used for preclinical FLASH irradiations.^13^ So far, there are no reports of an MLFC for therapeutic electron beams or an MLFC for electrons with more than two plates.

In this work, we report on the development, calibration and tests of a compact, portable MLFC with 128 plates that has been optimized for electron energies in the range between 3 and 25 MeV. Unlike the MLFCs mentioned above, the charge from the plates is not measured permanently, but in between the beam pulses or after enough charge has accumulated on the plates to achieve a good signal‐to‐noise ratio. The measuring principle allows the device to be used for strongly pulsed electron beams as present in irradiations with ultra‐high doses per pulse for studies of the FLASH effect as well as for conventional clinical electron beams. The device is a further development that has evolved from an in‐house developed beam instrumentation MLFC mounted at the end of the 50‐MeV accelerator structure of PTB's research linac as replacement of a beam dump to measure the energy in real time during beam optimization.^53^ Compared to its predecessor, the new device is portable, optimized for the electron energies relevant in radiation therapy (thinner MLFC plates), and the new improved electronics (a magnitude better signal‐to‐noise ratio) can read out the relatively small charges from conventional therapeutic electron beams (3 nC/s, 8 pC per pulse), much smaller than at the beam dump of the research linac (1.5 μC per pulse). The portable MLFC can be also used in proton beams of up to 100 MeV. For higher energies, the range is so deep that the Bragg peak would be behind the last plate. Therefore, for higher energies, a range shifter with a thickness depending on the energy range to be measured is inserted in front of the MLFC. As proof of concept, the device was tested in proton beams from a synchrotron source. Unlike a cyclotron source, where a continuous beam of a few hundred picoampere to nanoampere^41^, ^44^, ^48^ can be used, a synchrotron produces ion beam pulses (spill). Here, the novel readout principle of the presented MLFC is advantageous, that is, to measure the accumulated charge on the plates after a spill was delivered instead of measuring permanently small currents.

The MLFC can be set up quickly and features a read‐out unit that displays the measured energy value in real time.

## 2 MATERIAL AND METHODS

### 2.1 MLFC design and operating principle

The transportable MLFC detector consists of a stack of 128 Al‐plates with 0.3 mm thickness and a size of 150 mm × 150 mm that are galvanically insulated from ground and from each other by 75 μm fiberglass. Thin metal layers of 18 μm Cu on earth potential between the Al‐plates shield the electric field from the charge stored in the adjacent Al‐plates and eliminate channel crosstalk. A thin insulating layer of 45 μm polyamide serves as galvanic insulation against the subsequent leaf. The insulation resistance of the Al plates is > 2 GΩ, meaning that the collected charge is stored virtually loss‐free until its readout. Thanks to this feature, it is not necessary to read out all 128 plates parallel (at the same time). Instead, signals can be acquired using a single charge integrator in combination with a multiplexer. This simplifies the design, as otherwise, 128 separate current integrators would need to be individually calibrated.

**FIGURE 1:**
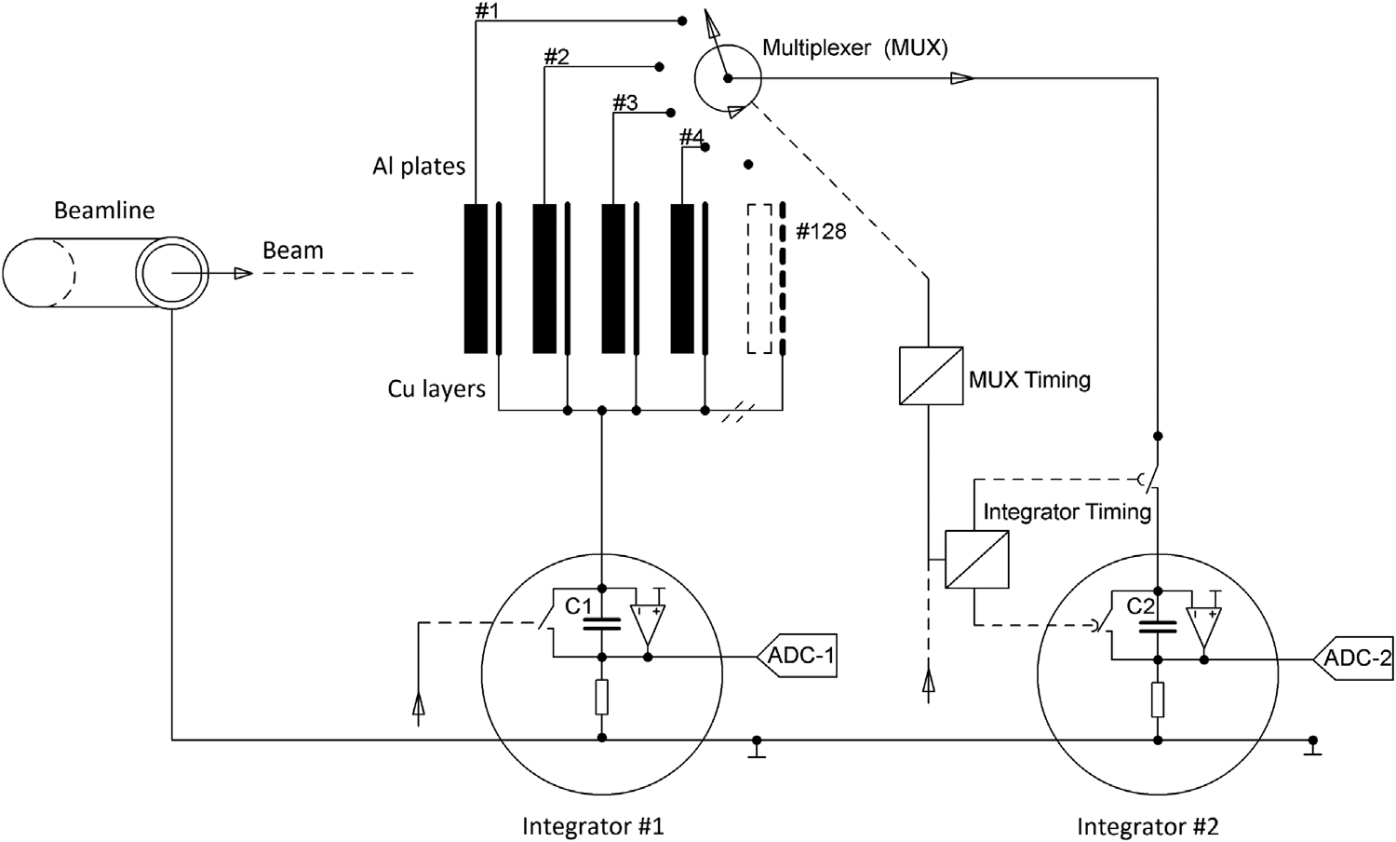
Schematic diagram of the MLFC detector.

Figure 1 shows a schematic diagram of the MLFC detector. The 128 galvanically insulated Al‐plates are perpendicular to the beam and act as the actual beam absorber. They are individually connected to one of the 128 inputs of a multiplexer and are thus isolated from the electronics during irradiation. The charge collected on an Al‐plate creates an image charge of equal magnitude but opposite polarity on the thin Cu layers on earth potential next to the charged Al‐plate. The formation of this image charge induces a current during the charge balancing recorded by charge integrator #1. All Cu‐layers are permanently electrically connected to each other and to the input of integrator #1. In this way, the total charge collected in all Al‐plates is constantly monitored without disturbing the characteristic charge distribution stored on the Al‐plates. Integrator #1 works bidirectionally, that is, acts as a charge sink during the formation of the image charge during the irradiation or as a charge source during the readout of the Al‐plates and the resulting vanishing of the image charge. The charge on the 128 Al‐plates is read out sequentially by charge integrator #2. The readout starts immediately after each beam pulse or after the total charge deposit in the MLFC, evaluated from the image charge measured by integrator #1, has exceeded a selectable threshold value. The Cu‐layers are much thinner than the Al‐plates. Therefore, the portion of the charge from the primary beam that stops in the Cu‐layers and contributes to the charge recorded by integrator #1 is negligible. When sequentially reading out the Al‐plates, the Cu‐layers at earth potential shield the Al‐plate from the electric field of the adjacent Al‐plate that is still charged. Thus, the grounded Cu‐layers between the Al‐plates avoid channel‐to‐channel crosstalk and enable a sequential read out.

The dimensions and total thickness of the leaf stack were chosen in such a way that the stack is large enough to absorb all primary electrons of clinical beams up to the highest usually available energy (24 MeV) and without scattered electrons leaving the detector laterally; on the other hand, the leaves are thin enough that the charge distribution from an electron beam with the lowest usually available clinical energy (4 MeV) is still well resolved by the first 15 MLFC channels.

### 2.2 MLFC signal acquisition

The readout electronics require about 90 ms to scan all 128 channels and the software in the readout unit needs about 10 ms to analyze the measured charge distribution and to display the energy value based on a previous experimentally determined calibration function (see below). This allows a pulse‐resolved measurement for up to 10 beam pulses per second. Three variants of integrators cover the measuring range from 100 pC to 10 μC (full scale); switching between three measuring ranges is possible in each case. After each beam pulse, the total pulse charge stored in the Al‐plates is evaluated based on the image charge at the Cu‐layers recorded by integrator #1. If the selected measuring range of integrator #2 is adequate, then the multiplexer starts to pass the individual detector channels to integrator #2 for readout. The image charge measured by means of integrator #1 decreases proportionally to the discharge of the Al‐plates during readout. Finally, the detector is discharged before the subsequent beam pulse. The energy is displayed in real time on the screen of the MLFC read‐out unit and pulse resolved recorded.

An integrating mode can be used if the pulse charge is below the detection limit (<1 nC per pulse). During the irradiation, the entire charge deposited in the detector is continuously monitored by integrator #1 without distorting the energy‐specific charge distribution. The readout starts after an adjustable threshold for the charge recorded by integrator #1 is reached or after a predefined time span.

For beams with pulse repetition frequency ≤10 Hz, the MLFC is read out between the beam pulses. For beams with pulse repetition frequencies >10 Hz, pulse trains are integrated and the readout starts subsequently.

The specifications of the MLFC are summarized in Table 1.

**TABLE 1:** MLFC specifications.

Number of channels	128
Leaf material	0.3 mm Al
	75 μm fiberglass
	18 μm Cu
	45 μm polyamide
Charge integrators	2
Measuring ranges	100 pC to 10 μC full scale
	(three ranges selectable)
Noise level	10 pC peak‐to‐peak
Readout time	100 ms
Pulse resolved reading	≤10 Hz
Pulse charge range	1 nC to 10 μC
Electron energy range	3–25 MeV
Proton energy ranges	Up to 100 MeV
with 3 cm Al	90–165 MeV
with 9 cm Al	165–225 MeV
with 15 cm Al	225–270 MeV
Detector dimensions	170 × 170 × 70 mm; 4 kg
Patent Pub. No.	US 11,933,925B2

### 2.3 Measurement setups

The portable MLFC was calibrated with monoenergetic electron beams at the exit of a 180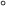 magnetic spectrometer^54^ at PTB's research LINAC.^55^ The beam energy of this LINAC can be varied continuously (up to 50 MeV). The beginning of the beam line is an electromagnet for separation of quasi mono‐energetic electron beams. The measured energies of the beams used for the MLFC calibration amount to 3.55, 5.71, 10.48, 15.21, 20.00, and 25.34 MeV. The full‐width at half‐maximum (FWHM) energy spread is between 0.02 MeV (at 3.55 MeV) and 0.18 MeV (at 25.34 MeV). The charge per beam pulse was set to a few nanocoulomb. The beam exit window after the 180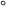 magnetic spectrometer is a 50‐μm brass foil. The effect of the energy loss of the primary electron beam due to the exit window on the MLFC was investigated by placing a second brass foil of the same type in between the spectrometer exit window and the MLFC.

**FIGURE 2:**
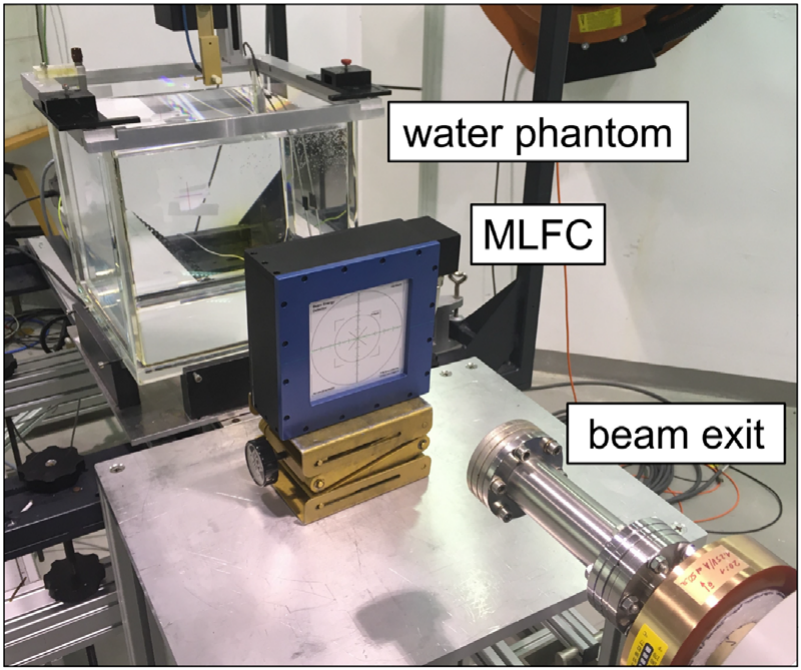
Measurement setup with the MLFC in front of the beam exit of PTB's research LINAC. The water phantom was used to measure the dose per pulse by means of alanine when the MLFC was removed.

In order to estimate the energy resolution of the MLFC, the device was positioned in front of the beamline of PTB's research LINAC (Figure 2) and it was tested if the 53‐keV energy loss caused by a 30‐μm Cu foil or the 13‐keV energy loss due to a 25‐μm Al foil in a 20‐MeV beam can be resolved.

**FIGURE 3:**
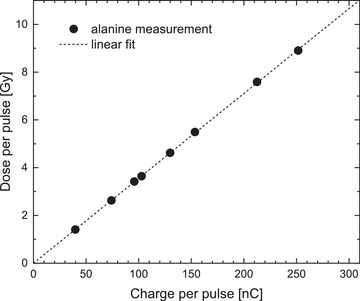
Dose per beam pulse measured by means of alanine in the water phantom as function of the charge per pulse measured by means of the ICT beam monitor. Error bars smaller than symbols: 0.015 nC for pulse charge and 0.7% for dose (k=1). Residuals from linear fit <0.5%.

In order to test the MLFC under ultra‐high dose per pulse conditions, the detector was used in the setup for PTB's ultra‐high pulse dose rate reference electron beam^55^ in front of the water phantom used for dose measurement (see Figure 2). The charge per beam pulse was varied from 30 to 320 nC by the variation of the pulse current at a constant pulse duration of 3 μs by changing the width of a slit diaphragm at the beginning of the beamline as well as by varying the pulse duration (0.1–3 μs) at a constant pulse current. The charge per beam pulse was measured nondestructively^56^ by means of an integrating current transformer (ICT, Bergoz Instrumentation). The corresponding dose per beam pulse in the water phantom was measured by means of PTB's alanine dosimetry system.^57^ The entrance window of the water phantom was placed 70 cm in front of the beam exit window. This is a typical setup for testing dosimeters for FLASH radiotherapy^58^, ^59^, ^60^ at PTB's ultra‐high pulse dose rate reference electron beam.^55^ Alanine pellets (5mm diameter, 3 mm height) were irradiated with 20 MeV at different charge per pulse values in a PMMA tube placed in the water phantom at reference depth (4.65 cm). The measured absorbed dose‐to‐water as function of the charge per pulse (Figure 3) was used to convert the ICT signal to the corresponding dose per pulse when the beam is intercepted by the MLFC. The used pulse repetition frequency was 5 Hz, the MLFC was operated in pulse resolved mode.

**FIGURE 4:**
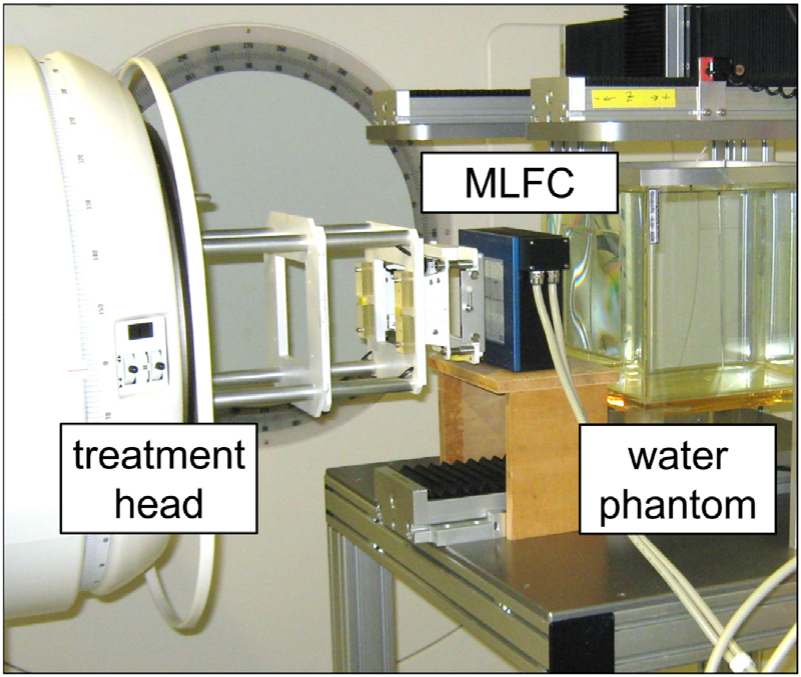
Measurement setups with the MLFC in front of the electron applicator of a medical LINAC. The water phantom was used to record PDD curves after the MLFC was removed.

In a further experiment, the MLFC was positioned in front of the treatment head (horizontal beam) of a medical LINAC (Elekta Precise) as shown in Figure 4 for measurement at conventional dose rates (4 Gy/min). Here, the MLFC was operated in integrating mode. The charge was integrated for about 10 s before the charge distribution stored in the MLFC was read out. The nominal energies used were 4, 6, 8, 10, 12, 15, and 18 MeV. After the MLFC was removed, the corresponding PDD curves in a water phantom were measured by means of a microDiamond detector as well as an ionization chamber to relate it to the recorded MLFC charge distributions.

Furthermore, the portable MLFC system was also tested for clinical ion beam applications in collaboration with the ion beam therapy center MedAustron in Wiener Neustadt. The beam source is a synchrotron that can produce proton or carbon beams with 5 s duration (spill). The typical charge within a single spill is about 1 nC. As proof of concept, MLFC measurements were performed with only one spill for each energy. This represents the application scenario for treatment, that is, one spill per depth layer (per energy) during layer‐by‐layer scanning. The MLFC was positioned at about 1 m in front of the beam nozzle. Proton beams with nominal energies of 100.4, 120.2, 140.8, 159.0, 200.7, 220.0, and 252.7 MeV were used. The nominal energies relate to the energy in the vacuum of the beam line estimated from the current at bending magnets. The energy decreases when it exits the vacuum through the nozzle due to the interaction with several layers of different material. The energy loss determined using a fully validated MC model of the nozzle^61^ ranging from 2.3 MeV at 100.4 MeV to 1.4 MeV at 252.7 MeV. From 100.4 to 159.0 MeV, a 3‐cm Al block was placed in front of the MLFC as range shifter in order to achieve, that the protons stop within the MLFC. For 200.7 and 220.0 MeV, a 9‐cm Al block was used, while for 252.7 MeV, the Al block was 15 cm thick.

### 2.4 MC simulations

Simulations were carried out using FLUKA (Version 4‐2.1, https://fluka.cern)^62^, ^63^ with 10e+05 primary particles and five cycles. In the proton beam model, a monoenergetic parallel proton beam with a Gaussian cross‐section (5 mm FWHM) propagates in air through an Al‐block (3 cm Al at 100.4, 120.2, 140.8, and 159 MeV, 9 cm Al at 220 MeV and 15 cm Al at 252.7 MeV) into the MLFC. In the electron beam model, monoenergetic parallel electron beams with a Gaussian cross‐section of 4 mm FWHM in the vacuum of the beam line pass through a 50‐μm brass foil exit window and enter the MLFC. Such electron beam model for the research accelerator has shown to be in good agreement with measurements of beam size and depth dose.^55^

The charge distribution in the MLFC is simulated by a 15 × 15 × 5.606 ${\mathrm{cm}}^{3}$ cube consisting of 512 alternating layers of aluminum, fiberglass, copper, and polyamide with thicknesses as given in Table 1. The experimental determination of the average density of a leaf consisting of these different materials by accurate determination of the weight (25.4505 g) and the accurately measured thickness (434.4 μm) resulted in 2.569 g/${\mathrm{cm}}^{3}$, a deviation of −5.1% compared to the value calculated based on manufacturer's data for thicknesses and the standard values for the densities. The manufacturer does not provide any information on the density of the fiberglass. Since fiberglass as a composite material of glass fiber and epoxy can have much smaller density than pure glass fiber, the density for fiberglass was reasonably adjusted so that the average density in the MC model matches the actual density.

A FLUKA USRBIN mesh with a resolution of 0.044 cm in the beam direction was used.

## 3 RESULTS

### 3.1 Electron energy calibration

**FIGURE 5:**
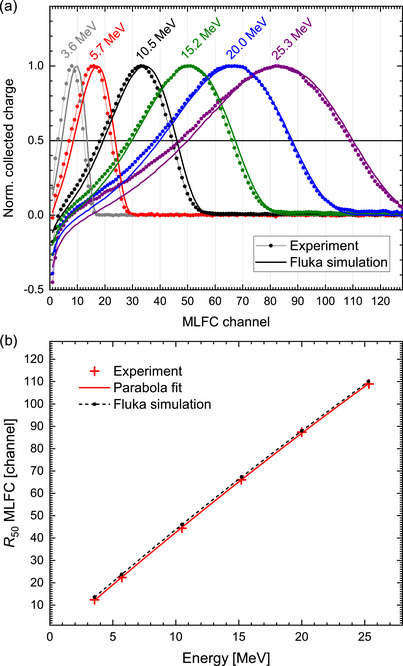
(a) Charge distributions measured by means of the MLFC normalized to the maximum for monoenergetic electron beams with different energies from measurements by means of the magnetic spectrometer as indicated. Thick lines: results from FLUKA simulations for the same energies as used in the experiment. (b) Half‐value depth $R_{50}$ of the charge distributions as a function of the beam energy.

The charge distributions of monoenergetic electron beams are recorded by means of the MLFC as a function of the beam energy measured by means of the magnetic spectrometer. Figure 5a shows the charge distributions of the MLFC normalized to the maximum for the different energies. The position of the maximum and half‐value depths $R_{50}$ (i.e., the depth at which the charge drops to 50%) increases with the energy. For higher energies, the charge distributions exhibit negative values in the first channels. This is due to the fact that more secondary electrons are “kicked” from the first aluminum plates to subsequent plates due to collisions with primary electrons than the number of primary electrons that stop in the first plates. All these features can also be observed in the charge distributions from the MC simulations of electron beams in pure aluminum carried out by Andreo et al.^49^ and our FLUKA simulations of the complex MLFC for the same energies as used in the experiment (thick lines in Figure 5a). A disagreement is evident for 25.3 and 20.0 MeV at lower MLFC channels. There is a fraction of scattered electrons with lower energy and shorter range that are not correctly reproduced by the MC model. However, this has no effect on the agreement of the $R_{50}$ value. All simulated curves are shifted by about one channel to larger channels. This means that the density of the plates is slightly higher than assumed.

As shown in Figure 5b, the half‐value depth $R_{50}$ increases almost linearly with the energy. Due to increasing stopping power in this energy range (electrons in Al), the function is slightly sublinear. The solid red curve is a parabola fit that serves as a calibration function. For comparison, the $R_{50}$ values from the MC simulation are shown as dashed black curve. The offset between the curves amounts to only about 1.2 channels. The leaf material comes from industry and is actually intended for a different purpose. Purity of the materials and actual density of the fiberglass and the polyamide layer are not known. The thickness of the layers follows the manufacturer's specifications. Given these considerable uncertainties for the MC model, the agreement of experiment and simulation is good. Only the experimental curve is used as calibration function.

**FIGURE 6:**
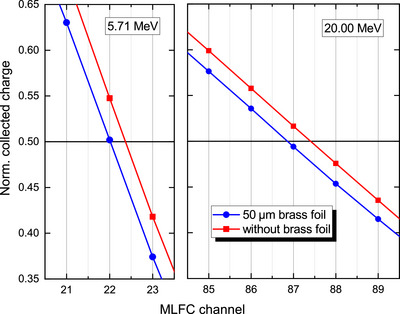
Section around half‐value depth $R_{50}$ of charge distributions from the MLFC normalized to the maximum with and without an additional 50‐μm thick brass foil between beam exit and MLFC for 5.71 and 20.00 MeV.

In order to determine the effect of the 50‐μm brass foil in the beam exit window, a piece of the same brass foil was additionally placed between the exit window and the MLFC. Then charge distributions were recorded at the six different energies. As shown in Figure 6, a shift in the $R_{50}$ values to lower MLFC channels, 0.35±0.05 channels for 5.71 MeV and 0.55±0.05 channels for 20.00 MeV, can be clearly observed. Using the calibration function (solid red curve in Figure 5b), the corresponding energy losses are determined to be (79±11) keV and (122±11) keV, respectively. This agrees with the theoretical values according to the stopping power (50‐μm CuZn5 at 5.71 MeV: 74 keV, at 20 MeV: 122 keV). The energy loss in the beam exit window is taken into account in the calibration function by theoretical values.

**FIGURE 7:**
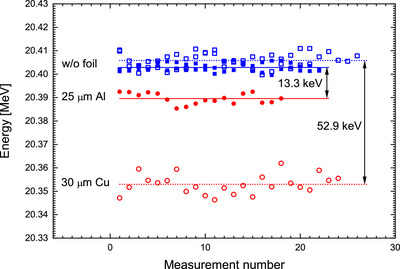
Reading of the MLFC with (red circles) and without (blue squares) a 30‐μm-thick Cu foil (open symbols) or a 25‐μm-thick Al foil (full symbols) placed between beam exit window and the MLFC. Differences of mean energies (lines) are indicated.

The distinct shift in the $R_{50}$ values due to 122 keV energy loss by a brass foil suggests a high energy sensitivity. In order to estimate the energy resolution of the MLFC, the effect of even thinner foils was tested. The MLFC was positioned in front of the beamline of PTB's research LINAC (Figure 2) and irradiated with 20‐MeV electrons with and without a 30‐μm Cu foil, as well as a 25‐μm Al foil between the beam exit window and the MLFC. About 20 measurements of about 50 beam pulses were taken without foil, then with foil, and then again without foil (Figure 7). There was no significant difference in the MLFC energy measurements before and after the measurement at each foil. With the 30‐μm Cu foil, where a theoretical energy loss of 52.6 keV is expected, a difference of (52.9±0.9) keV was measured. For 25 μm Al, the theoretical value is 13.5 keV, and (13.3±0.7) keV was measured. This suggests that energy changes due to fluctuations of a LINAC of 20 keV (0.1% of 20 MeV) can be identified in a single measurement during the QA using such MLFCs.

### 3.2 Ultra‐high dose per pulse electrons

The MLFC was tested in the setup for PTB's ultra‐high pulse dose rate reference electron beam^55^ in front of the water phantom used for dose measurement (Figure 2) and irradiated with different beam intensities, that is, different charges per beam pulse. The current of the magnet for separation of mono‐energetic electron beams and thus the beam energy was left constant. The corresponding dose per pulse at the reference depth in the water phantom was determined by means of PTB's alanine dosimetry system (Figure 3). The dose per pulse was in the range of 1–10 Gy and thus in the ultra‐high dose per pulse range as typically used in studies of the FLASH effect.^4^

**FIGURE 8:**
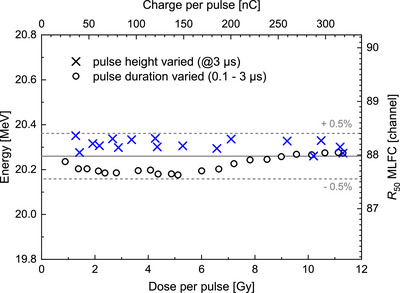
Energy (left *y*‐axis) determined from the half‐value depths $R_{50}$ of the MLFC charge distributions (right *y*‐axis) of the electron beam from PTB's research LINAC as a function of the charge per pulse (upper *x*‐axis) and corresponding dose per pulse in the water phantom of the setup for ultra‐high pulse dose rates (lower *x*‐axis).

Using the determined calibration function (solid red curve in Figure 5b), the beam energy is determined from the half‐value depths $R_{50}$ of the recorded charge distributions. As shown in Figure 8, the beam energy can be measured with precision (±0.5%) independent of the dose per pulse. Since the energy is determined in real time by the MLFC readout unit, it was possible to record the data series shown in Figure 8 in a few minutes.

### 3.3 Conventional dose rate electrons

**FIGURE 9:**
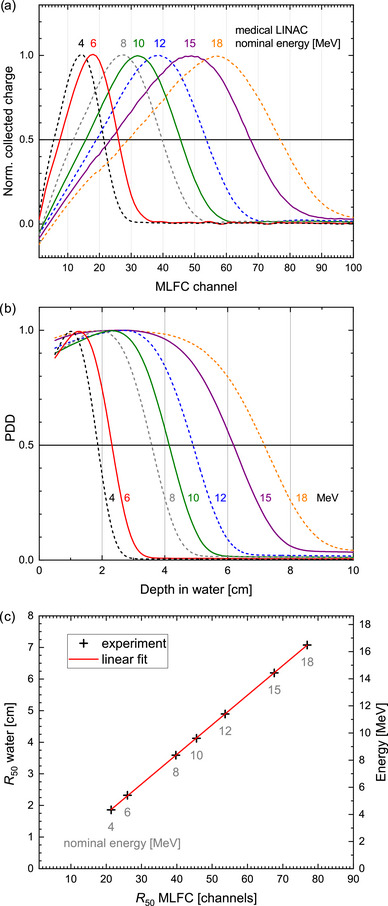
(a) Charge distribution measured by means of the MLFC for electron beams from a medical LINAC (3–4 nC/s) of different nominal energies as indicated. Maximum corresponds to 220 pC (4 MeV) to 105 pC (18 MeV). (b) PDD curves measured in water under same conditions with microDiamod detector and ionization chamber. (c) Half‐value depth $R_{50}$ from the PDD measurements in water as function of half‐value depth of the corresponding MLFC charge distributions, right *y*‐axis: energy calculated from $R_{50}$ water (see text).

The MLFC was also tested in beams with conventional dose rates from a medical LINAC (Figure 4). Figure 9a shows MLFC charge distributions for electron beams with nominal energies from 4 to 18 MeV, and Figure 9b shows the corresponding PDD curves measured in the water phantom under the same conditions after the MLFC was removed. Each PDD curve was recorded using a microDiamod detector (PTW T60019) as well as with a Roos ionization chamber (PTW T34001) following the instructions in IAEA TRS398.^64^ Both agreed in each case. Figure 9c shows the half‐value depth $R_{50}$ of the PDD measurements in the water phantom as function of the corresponding half‐value depth of the MLFC charge distributions. Although dose (in water) and charge (in Al plates) are different things, a linear relationship is observed (red line). If one relates the theoretical ranges of electrons in water to the ranges in Al, then in the energy range from 4 to 18 MeV, the deviations from a linear relationship are less than ±1%. And if $R_{50}$ of PDDs from MC simulations^65^ is plotted versus $R_{50}$ of charge distributions in pure Al for 5, 10, and 20 MeV from MC simulation,^49^ the deviations from linear relationship is also less than ±1%. Using the linear fit shown in Figure 9c as calibration function, the half‐value depth $R_{50}$ in water can be displayed in real time on the screen of the MLFC read‐out unit. The mean energy at the surface of the water phantom $E_{0}$ can also be displayed using the recommended relationship $E_{0}=0.656+2.059R_{50}+0.022{\left(R_{50}\right)}^{2}$, where $R_{50}$ in water is given in cm and $E_{0}$ is given in MeV.^66^ The right *y*‐axis of the diagram in Figure 9c shows the energy according to $E_{0}=2.33R_{50}$, which is a common linear approximation for the aforementioned function. The nominal energies differ from the measured energies by up to ±10%, which is normal for a medical LINAC.^67^

### 3.4 Proton beams

**FIGURE 10:**
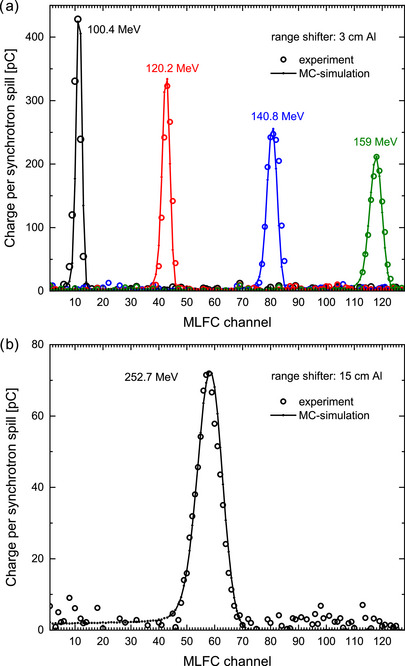
Charge distributions from MLFC measurements of proton beams for a single spill of about 1 nC each for different nominal energies as indicated using 3 cm (a) or 15 cm (b) aluminum as a range shifter in front of the MLFC. Curves represent results from FLUKA simulations where the input parameter “energy” was optimized until agreement with the measurements was achieved (see Table 2).

**FIGURE 11:**
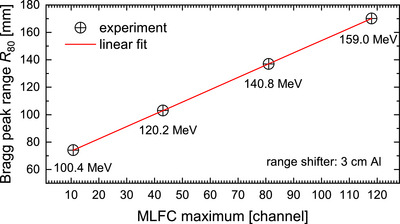
MLFC calibration function for determination of the Bragg peak range $R_{80}$ in water from the maximum of MLFC charge distribution.

The circles in Figure 10 represent experimental charge distributions from measurements at MedAustron in proton beams with therapeutic energies for only one single synchrotron spill for each energy. The total charge per spill was between 0.8 and 1.2 nC. The measured depth–charge curves exhibit well‐defined peaks. The curves in Figure 10 represent the results of MC simulations where the input parameter “energy” (Section 2.4) was optimized until a good agreement with the measurements was reached. Table 2 compares the energy after the beam exits the nozzle with the results of the MC simulation of the MLFC measurements. Deviations of about −2% are observed, which are attributed to the uncertainty of the MLFC model, in particular, due to the uncertainties of the thickness and density of the insulator layers. However, the MC model is irrelevant for the application. Since the range in water must be verified during QA, the MLFC is calibrated against the positions of the Bragg peak in a water phantom. These are routinely measured to ensure that they are the same as when the irradiation room was commissioned. Table 2 gives the measured $R_{80}$ ranges for the used energies and the positions of the maxima of a Gaussian fit to the measured MLFC charge distributions. Figure 11 shows as example the resulting calibration function for the case using the 3‐cm Al rage shifter. The residuals from the linear fit are <0.03 mm (<0.02%).

**TABLE 2:** Deviation of energy from MC simulation of measured MLFC charge distribution from energy taking into account the energy loss in the nozzle from MC simulation,^61^, ^62^ as well as the range of the Bragg peak ($R_{80}$) measured in a water phantom and position of the maximum in the measured MLFC charge distribution (Gaussian fit).

Nominal energy	Energy after nozzle from MC	Range in water $R_{80}$	MLFC maximum	Energy from MC of MLFC	Deviation
[MeV]	[MeV]	[mm]	[channel]	[MeV]	[%]
100.4	98.1	74.1	10.79	96.3	−1.8
120.2	118.2	103.0	43.01	115.9	−1.9
140.8	139.0	137.0	81.02	136.4	−1.9
159.0	157.3	170.2	117.98	154.5	−1.8
200.7	199.4	256.2	66.67	195.0	−2.2
220.0	218.7	300.2	115.38	213.7	−2.3
252.7	251.3	380.1	57.77	245.5	−2.3

As the energy increases, the width of the MLFC charge distributions becomes broader, in agreement with the simulation. At a nominal energy of 252.7 MeV, the total charge of the spill of 0.8 nC is distributed over 20 channels; the maximum of the distribution thus amounts to only 72 pC (Figure 10b). Nevertheless, the peak is well defined, and its position can be evaluated well thanks to the low noise level of only about 10 pC peak‐to‐peak. The noise level can be observed well via the scattering of the data points in channels >80, which the beam does not reach and where the charge should be zero. The low energy tail visible in the simulated charge distribution in channels <45 is attributed to charged secondary particles from inelastic nuclear interactions of the protons.^36^, ^37^

## 4 DISCUSSION

By means of the portable MLFC, the energy of an electron beam can be determined quickly and effortlessly. The MLFC detector is placed in front of the accelerator, and the read‐out unit displays the determined energy value in real time. We demonstrated that the MLFC works well in beams with ultra‐high pulse dose rates as well in the conventional beams from a medical LINAC. An energy variation of about 13 keV was clearly resolved in the MLFC measurements. This suggests that energy changes of 0.1% for a 20‐MeV beam can be detected during the QA by means of the MLFC. Rapid validation of energy stability may prove to be of particular importance for irradiation experiments in which the FLASH effect is investigated, in particular when using prototype LINACs or emerging commercially available FLASH electron accelerators, which have no electromagnet for the selection of a defined energy. Thus, the energy depends on the used radio frequency power and the amount of charge from the electron gun (beam loading effect) which is changed when the pulse duration or beam current is varied (either intentionally or unintentionally). The MLFC allows the energy to be measured quickly before and after irradiation without time‐consuming recording of a PDD curve, which may present, in the case of ultra‐high dose‐rate beams, a non‐negligible radiation protection problem. This could be particularly useful when comparing irradiation with conventional (low) and ultra‐high dose rates, or when preparing a beam for the repetition of FLASH irradiation experiments with the same beam characteristics as those previously used.

As proof of concept, the same MLFC system was used in therapeutical proton beams using range shifters with different thicknesses. The measured depth‐charge curves exhibit well‐defined peaks despite the fact that only the charge of one single synchrotron spill of about 1 nC was collected. MC simulations describe the experimental data well. It is possible to calibrate the channel position of the peak in the charge distributions against the position of the Bragg peak of the corresponding PDD curves and use this function for the real‐time display of the readout unit. Measuring larger proton currents, as required for ultra‐high dose rates for FLASH with protons, would be even advantageous, since more charge per spill will improve the signal‐to‐noise ratio.

## 5 CONCLUSION

In summary, the MLFC can be used for rapid validation of energy stability when carrying out experiments comparing electron beams of conventional and ultra‐high dose rates. The same device can be used in the pulsed therapeutical ion beams from a synchrotron.

## CONFLICT OF INTEREST STATEMENT

The authors declare no conflicts of interest.
